# Outcome of ankle fractures in children: an updated systematic review and meta-analysis

**DOI:** 10.1308/rcsann.2025.0124

**Published:** 2026-02-02

**Authors:** N Badhe, K Ferdinand, J Chilaka, RY Yap, C Durand, BA Marson

**Affiliations:** ^1^ University of Nottingham, UK; ^2^ Cambridge University, UK; ^3^ Alder Hey Children’s NHS Foundation Trust, UK; ^4^ Health Education England North West, UK; ^5^ University Hospitals of North Midlands NHS Trust, UK

**Keywords:** Ankle, Children, Fracture, Injury, Malleolus, Paediatric

## Abstract

**Introduction:**

Low-risk ankle fractures in children are commonly managed with immobilisation, using options such as supportive bandages, removable boots/braces or casted devices. The aim of this study was to review the literature to determine the optimal treatment for these fractures.

**Methods:**

A systematic review of randomised controlled trials (RCTs) was conducted in November 2024. Comprehensive searches of MEDLINE, Embase and Cochrane CENTRAL were performed. Studies recruiting children (0–18 years) with low-risk, stable or suspected occult ankle fractures managed non-operatively were included, comparing different immobilisation devices. Unscheduled returns to healthcare facilities, patient-reported outcomes, functional outcomes, and complication rates were assessed.

**Findings:**

Seven RCTs involving 503 children were included. Compared with casts, removable boots/braces significantly reduced unscheduled healthcare visits (risk ratio [RR] 0.26; 95% confidence interval [CI] 0.13 to 0.52; *p* = 0.0001), shortened return to normal activities (mean difference [MD] −6.83 days; 95% CI −11.20 to −2.96; *p* = 0.0031), and improved functional scores (standardised mean difference [SMD] −0.79; 95% CI −1.03 to −0.55; *p* < 0.00001). Patient and parent satisfaction was also higher with removable devices. Supportive bandaging reduced missed school days (MD −0.49; 95% CI −0.81 to −0.18; *p* = 0.002) and accelerated return to baseline activities (14.17 vs 20.19 days; *p* = 0.0002), although results showed high heterogeneity. No significant differences were found in pain, complications, emotional wellbeing or mobility between groups.

**Conclusions:**

Removable boots or braces offer a favourable alternative to cast immobilisation for low-risk paediatric ankle fractures. However, the necessity of rigid over soft immobilisation remains uncertain. A definitive RCT based on a core outcome set and cost-effective analyses is required to inform treatment recommendations.

## Introduction

Ankle fractures are a common cause of injury in children, with an estimated incidence of 1 in 1,000 children per year, accounting for approximately 5% of all paediatric fractures.^1–3^ Paediatric ankle fractures can generally be divided into two broad categories: ‘high-risk’ fractures, which involve significant displacement and ‘low-risk’ fractures, which include occult fractures, undisplaced Salter–Harris type 1 and 2 fractures and avulsion fractures.^4,5^ Although it is generally agreed that high-risk ankle fractures require operative reduction for a good outcome,^1,6^ the optimal treatment for low-risk ankle fractures remains uncertain. This lack of consensus means there is significant variability in clinical practice, particularly regarding immobilisation devices used and the duration of treatment.^7,8^

A variety of treatment devices are commonly used in the management of low-risk, stable or occult ankle fractures, including both soft and rigid immobilisation devices.^5,9,10^ Soft (functional) immobilisation involves no formal immobilisation beyond a supportive bandage, in addition to ice and elevation. This facilitates early mobilisation but may cause greater initial pain.^11,12^

Rigid immobilisation devices can be divided into two main categories: removable boots or braces and casted devices. Removable boots and braces enable bathing and can be taken off at home, enhancing both hygiene and comfort.^4^ Walker boots provide support and mobility, allowing protected weight-bearing in low-risk paediatric ankle fractures.^3,13,14^ Similarly, ankle braces provide moderate support to the ankle, with some designs permitting dorsiflexion and plantarflexion.^4,15^

There are two main types of casted devices: custom-moulded splints and cast immobilisation. Custom-moulded casted splints are typically made from lightweight casting materials, secured with Velcro straps, enabling easy removal for bathing, but resist ankle dorsiflexion and plantarflexion.^15,16^ By contrast, cast immobilisation offers maximum immobilisation and protection.^10^ Casts can be weight-bearing or non-weight-bearing and may improve pain control but limit early motion.^4,12^ However, cast immobilisations are non-removable and have been associated with pressure-related complications and reduced bone mineral density in adolescents.^11,17^

In 2019, our team published a systematic review comparing treatments for low-risk ankle fractures. Since the original systematic review was published in 2019, several new randomised controlled trials (RCTs) have been published, and a core outcome set for childhood limb fractures has been established.^5,13,14,18,19^ This highlights the need for an up-to-date synthesis of the evidence using standardised, relevant outcomes to inform evidence-based treatment recommendations. The optimal treatment strategy for these injuries should support a safe and timely return to normal activities, while minimising the need for additional hospital visits for children and their parents.

The aim of this updated systematic review was to determine the best treatment for low-risk, stable and occult ankle fractures. The primary outcome was treatment failure, defined as an unscheduled return to the emergency department or orthopaedic clinic for additional treatment. Secondary outcomes assessed followed the framework set out in the core outcome set for childhood limb fractures. Cost-effectiveness, as well as both child and caregiver satisfaction, was also considered when evaluating treatment options.

## Methods

### Systematic literature search

A systematic review search was conducted in November 2024 to identify all peer-reviewed RCTs investigating the outcomes of different management strategies for low-risk, stable or suspected occult ankle fractures in children. This review was prospectively registered on the PRISMA database prior to undertaking the searches (CRD42024606237).

Searches were completed using OVID MEDLINE, OVID Embase and Cochrane CENTRAL databases using the search strategy shown in the supplementary materials. Publication date was restricted to 2017 to current because previous titles were screened in the original systematic review.^5^ Additional screening of grey literature was completed using Google Scholar.

### Inclusion criteria

Studies were included if they met the following inclusion criteria:

- reported outcomes of children (aged 0–18) with a low-risk, stable or suspected occult ankle fracture, treated within 48h of injury;
- compared the outcomes between different treatment devices provided to facilitate recovery from the injury;
- were RCTs or quasi-RCTs.

Non-randomised studies, biomechanical studies, animal studies, review articles and conference abstracts were excluded. In addition, studies investigating ankle fractures requiring surgical reduction or fixation and children with congenital malformations or genetic conditions that alter bone turnover or function were excluded.

Title and abstracting screening using the prespecified inclusion criteria was conducted independently by three reviewers (KF JC or RY). Full-text articles were then screened independently by two reviewers (JC and RY). Discrepancies between individual screening judgements were resolved through discussion or review by the senior author (BM).

### Data extraction

Data extraction from included studies was conducted independently by two reviewers (JC and RY) into a predesigned data extraction table. Discrepancies between independent extractions were resolved by consensus or senior author (BM) review. The following study characteristics were extracted: title; authors; year of publication; study site (country and type of institution); funding source; evidence of prospective registration; details of the intervention (device or treatment, duration of immobilisation and weight-bearing status); details of the control (device or treatment, duration of immobilisation and weight-bearing status); number of participants randomised per group; number included in the intention-to-treat analysis; and follow-up duration. For outcome data, mean and standard deviation (sd) values were extracted for continuous variables and number of events and denominator number were extracted for binary variables.

### Risk of bias

The risk of bias in included studies was assessed using the Cochrane Collaboration Risk of Bias 2 (RoB 2) tool.^20^ This is a validated instrument that assesses the risk of bias in RCTs across five bias domains: bias due to the randomisation process, deviation from the intended intervention, missing outcome data, measurement of outcomes, and selection of the reported result, and provides an overall risk of bias judgement. As before, two reviewers (JC and RY) independently assessed the risk of bias for each included study, with disagreements resolved by the senior author (BM).

### Outcomes

The primary extracted outcome was treatment failure, defined as a return to the emergency department or orthopaedic clinic for additional treatment. Secondary outcomes, which followed the core outcome set for childhood limb injuries included: pain and discomfort in the first 72h; return to physical and recreational activities; emotional and psychosocial wellbeing; complications from the injury and treatment; return to baseline activities of daily living (ADL); participation in learning; appearance and deformity; time to union; and recovery of mobility.^19^ Healthcare economic data and both patient and parent satisfaction scores presented in the included studies were also analysed.

### Statistical analysis

When considered appropriate, results of comparable RCTs were pooled. Statistical heterogeneity was assessed through visual inspection of forest plots, followed by calculation of the *I*^2^ statistic, with *I*^2^ > 75% considered as considerable heterogeneity, particularly if it could not be explained by the diversity of the methodological or clinical features of the studies. Mean and sd summary statistics were calculated using available metrics within included studies. If mean and sd were not available, medians, interquartile ranges and ranges were converted to mean and sd data using the formulae on MetaConverter (https://meta-converter.com/). Pooled data were analysed quantitatively via meta-analysis, performed in Review Manager 5, using the random-effects models. Mean difference (MD) or standardised mean difference (SMD) were used to compare continuous variables, and risk ratios (RRs) were used for binary variables. For outcomes only reported by one study, unpaired *t*-tests and chi-squared tests were performed using GraphPad Prism 10.3.1 to assess significance for continuous and categorical variables, respectively. Significance was set at *p* < 0.05.

## Findings

The PRISMA flow diagram for this study is shown in Figure 1. The initial search identified 1,518 studies, with 1,245 remaining after duplicates were removed. Title and abstract screening identified 23 potentially relevant studies, of which 7 met the inclusion criteria (Table 1). No additional studies were included through manually searching the references of included studies. The 7 included studies involved 503 patients with a mean age of 11.0 years.

**Figure 1:**
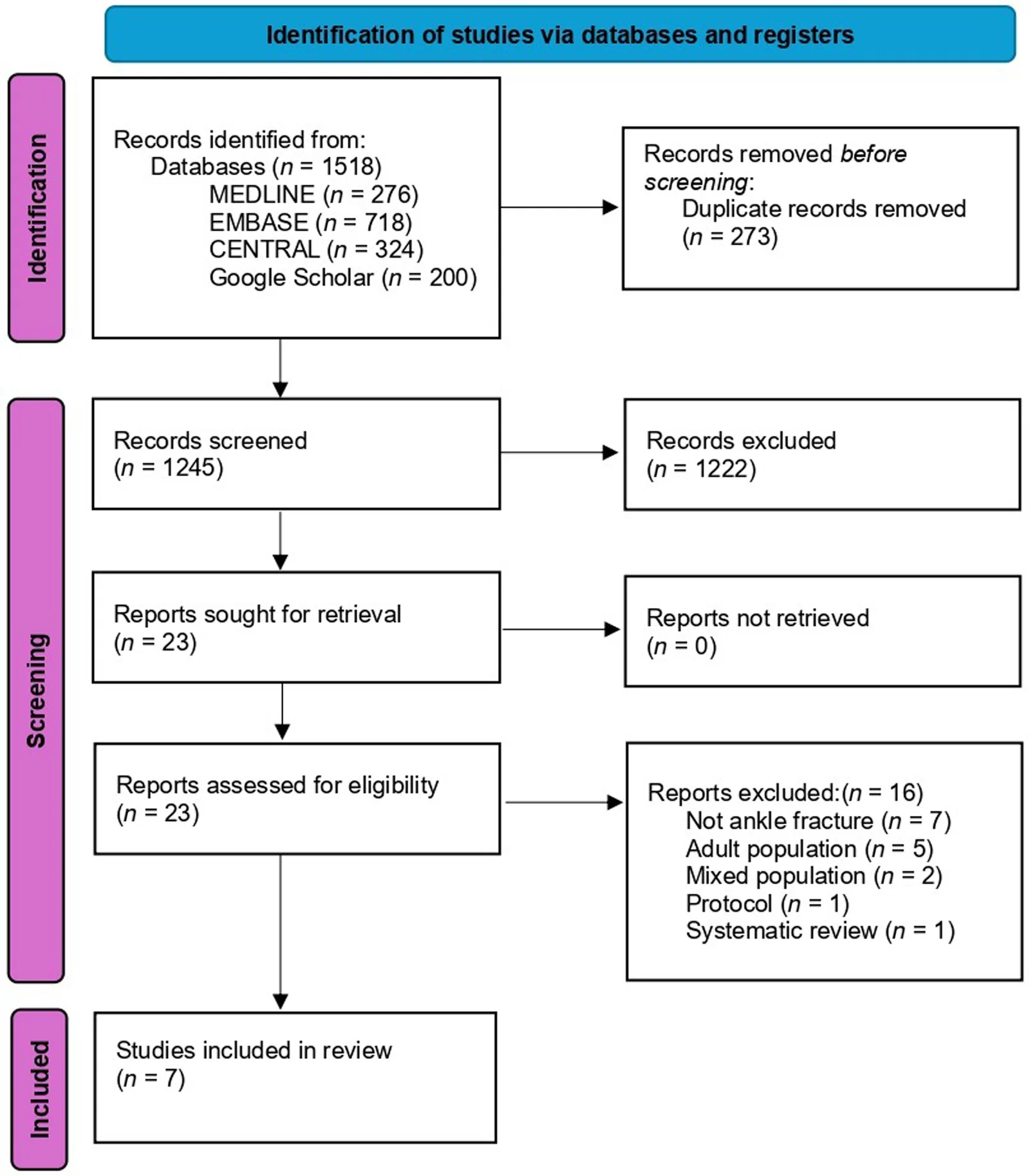
PRISMA flow diagram

**Table 1:** Demographics of patients, research characteristics, and primary outcomes reported for included studies

Study	Year	Setting	Participants			Injury	Intervention			Primary outcome
			(*n*)	Age range	Mean age		1	2	3	
Gleeson *et al*^12^	1996	Children’s hospital	45	Unspecified	9	Ankle injury with an assessment score >10 including swelling, growth plate tenderness and inability to bear weight	Tubular bandage and crutches, WBAT	Below-knee plaster cast, WBAT, cast off at 2 weeks	n/a	Custom score
Boutis *et al*^4^	2007	Tertiary paediatrics ED	104	5–15	10	Non-displaced SH1 with a clinical diagnosis, SH2, avulsion	Air-stirrup ankle brace, 5 days NWB then WBAT, remove brace when pain allows	Below-knee fibreglass cast, 5 days NWB then WBAT, cast off at 3 weeks	n/a	Modified ASK-p score at 4 weeks
Launay *et al*^11^	2008	Children’s hospital	62	8–15	14	Ankle injury with no fracture on x-ray	1-week functional treatment	1-week plaster immobilisation	n/a	Number of days off school by 1 week
Barnett *et al*^15^	2012	Children’s hospital	45	5–15	10	SH1 with tenderness over growth plate and normal x-ray, avulsion lateral talus or lateral malleolus	Air-stirrup ankle brace. WBAT, for 2–4 weeks based on examination at 12–16 days	Removable casted splint, WBAT, for 2–4 weeks based on examination at 12–16 days	n/a	ASK-p score at 4 weeks
Beck *et al*^13^	2023	Children’s hospital	60	5–16	11.3	Non-displaced Salter–Harris 1 distal fibula fracture or non-displaced distal fibula avulsion fracture	Non-waterproof, short leg, weight-bearing fibreglass cast, WBAT	CAM boot, WBAT	n/a	n/a
Hampton *et al*^14^	2024	Children’s hospital	100	6–16	11.2	Ankle sprains, small fragment avulsions, and bular Salter–Harris 1 injuries	Below-knee full synthetic cast for 4 weeks	Walker boot	n/a	Patient preferences between cast and walker boot treatment
Marson *et al*^18^	2025	Multicentre	87	5–15	11.4	Minimally or undisplaced distal fibula fracture or a clinical undisplaced ankle fracture	Supportive bandage worn for 2 weeks	Semi-rigid removable brace	Non-removable synthetic or resin-based below-knee walking cast	Treatment failure, defined as a return to the emergency department or orthopaedic clinic for additional treatment

ASK-p  =  Activity Scale for Kids performance scale; CAM  =  contolled ankle movement; ED  = emergency department; n/a  = not applicable; NWB  =  non-weight-bearing; SH1  =  Salter–Harris type 1 fracture; SH2  =  Salter–Harris type 2 fracture; WBAT  =  weight-bearing as tolerated

Risk of bias and data extraction for Marson *et al* was completed by reviewers independent of this study.^18^

### Risk of bias assessment

A risk of bias summary for the included studies is given in Table 2.

**Table 2:** Risk of bias summary for included studies

Study	D1	D2	D3	D4	D5	Overall
Gleeson *et al*^12^	L	L	M	L	L	L
Boutis *et al*^4^	L	L	L	L	L	L
Launay *et al*^11^	L	L	M	L	H	H
Barnett *et al*^15^	L	L	L	L	L	L
Beck *et al*^8^	L	L	L	L	L	L
Hampton *et al*^14^	L	L	L	L	L	L
Marson *et al*^18^	L	L	L	L	L	L

H  =  high; L  =  low; M  =  medium/some concerns

### Outcomes

#### Comparison 1: soft vs rigid immobilisation

Three trials investigated the outcomes in children with low-risk, stable or suspected occult ankle fractures treated with soft (bandage or no immobilisation) compared with rigid immobilisation (boot, brace or plaster).^11,12,18^

##### Pain

Pain at rest was reported by Launay *et al*, who identified no significant difference in pain experienced by patients in soft (functional) immobilisation and rigid plaster immobilisation at 1-week follow-up (1.1 vs 1.0; *p* = 0.1944).^11^

##### Emotional and psychosocial wellbeing: emotional/overall health

Emotional or overall health scores were pooled from two studies, showing no significant difference between soft and rigid immobilisation groups (SMD 0.06, 95% confidence interval [CI] −0.74 to 0.86; *p* = 0.88).^11,18^

##### Complications from injury and treatment

Skin complications during the study period were reported by one study.^18^ This study reported complications in six patients (6.9%): three (9.7%) in the walking cast group (reinjury, prolonged immobilisation, skin blister), two (7.4%) complications in the supportive bandage group (reinjury, persistent pain), and one (3.4%) in the removable splint group (patient required further immobilisation after removal).

##### Return to baseline ADL

Return to ADL was reported in two trials.^12,18^ Gleeson *et al* reported a significantly shorter mean time to return to normal activity in the Tubigrip supportive bandage group compared with the Plaster of Paris (POP) group (14.17 vs 20.19 days; *p* = 0.0002). Similarly, Marson *et al* reported the number of patients who returned to normal activities at 6-week follow-up was higher in the supportive bandage group (85.7%) compared with the rigid immobilisation group (67.7%). However, this difference was not statistically significant (*p* = 0.2069).

##### Participation in learning: days off school

Two studies reported participation in learning by measuring the number of school days children missed (Figure 2).^11,18^ Although only Launay *et al* found a significant difference between treatment groups, pooled analysis showed that soft immobilisation techniques resulted in significantly fewer days off school compared with rigid immobilisation, with a MD of −0.49 (95% CI −0.81 to −0.18; *p* = 0.002).

**Figure 2:**
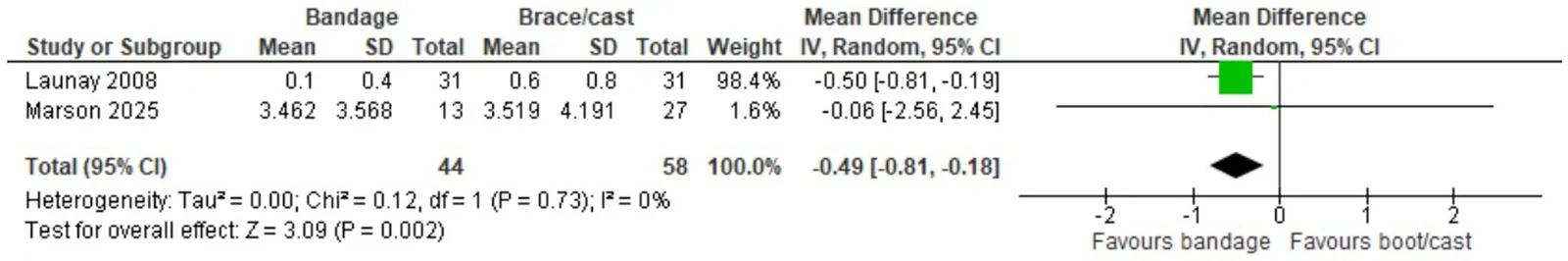
Forest plot comparing participation in learning between soft and rigid immobilisation groups.

##### Mobility

Mobility at 1 week following treatment implementation was reported by Launay *et al*, who assessed the proportion of children requiring assistance while walking.^11^ There was no significant difference between no immobilisation (6.5%) and rigid cast (19.4%) immobilisation groups (*p* = 0.1297).

Marson *et al* compared Proxy PROMIS mobility scores for patients randomised between supportive bandage or removable brace/cast groups.^18^ However, no significant difference was identified at 2-week (38.3 vs 37.5; *p* = 0.6809) or 6-week (52.3 vs 48.1; *p* = 0.0566) follow-up.

##### Healthcare economic cost

One trial reported the number of days of work parents of children missed.^11^ This study found that parents of children in the rigid cast immobilisation group missed significantly more days of work compared with those in the functional (no immobilisation) group (0.3 ± 0.8 vs 0.0 ± 0.0 days; *p* = 0.04).

##### Composite/assessment score

Gleeson *et al* reported a composite assessment score, measuring several domains including pain, swelling, growth plate tenderness and the degree of weight-bearing between supportive bandage and POP groups.^12^ However, this study found that differences between groups were not statistically significant at either 2-week (5.0 vs 6.3; *p* = 0.0522) or 4-week (3.4 vs 3.8; *p* = 0.2185) follow-up.

### Missing outcomes

None of the three trials comparing soft and rigid immobilisation devices reported on the primary outcome (unscheduled returns to a healthcare facility), or return to physical and recreational activities, appearance and deformity, or time to union, which are key outcomes from the core outcome set for childhood limb fractures.^19^

#### Comparison 2: boot/brace vs POP rigid immobilisation

Five trials investigated outcomes in children with low-risk, stable or suspected occult ankle fractures treated with rigid mobilisation, comparing the use of a removable boot/brace with a POP cast.^4,13–15,18^

##### Primary outcome: unscheduled returns to a healthcare facility

Unscheduled visits to a healthcare facility for immobilisation-related complications were reported in four trials (Figure 3).^4,13–15^ Three of these studies found a significantly higher rate of unscheduled returns in the cast group, whereas Barnett *et al* (25.0%) reported similar rates between treatment groups.^15^ Pooled analysis showed that removable boot/brace immobilisation devices were associated with significantly fewer returns to healthcare facilities, with a relative risk of 0.26 (95% CI 0.13 to 0.52; *p* = 0.0001).

**Figure 3:**
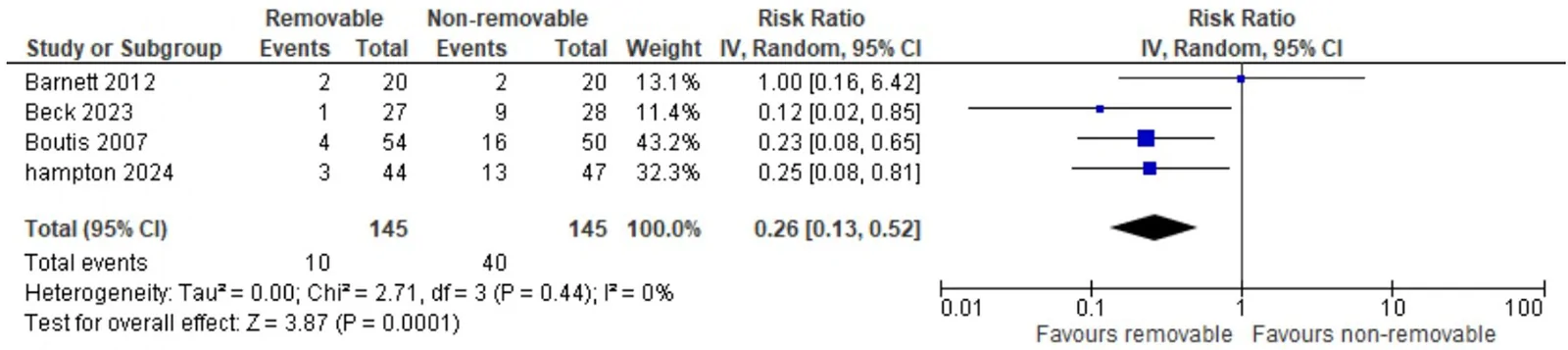
Forest plot comparing the need for unscheduled returns to a healthcare facility between boot/brace and casted device groups.

##### Pain

Pain scores were pooled from three studies, showing no significant difference between treatment groups (SMD −0.12, 95% CI −0.41 to 0.16; *p* = 0.39).^4,13,14^ One study reported the number of days of analgesia use over the first two weeks after immobilisation with an ankle brace compared with a casted splint. There was no difference in mean days of analgesia use, with a MD of 0.50 (95% CI −2.92 to 1.92; *p* = 0.6785).

##### Return to physical and recreational activities

Beck *et al* reported the ability of children to return to sport.^13^ This study found that 16 (29%) patients failed to return to sports at that time at 8-week follow-up, reporting no significant difference between fibreglass cast and walker boot groups. Reasons for children not returning to sports in this study included COVID-19 restrictions (9.1%), nervous about returning (7.3%), not involved in sports (5.5%), new onset pain (1.8%) and unknown (5.5%).

##### Emotional and psychosocial wellbeing: emotional/overall health

The emotional or overall health of children between removable and non-removable immobilisation groups was reported by two studies.^13,18^ Although both studies reported higher emotional/overall health scores in favour of removable boot/brace devices, the difference was only significant in Beck *et al* and pooled analysis demonstrated no significant difference between treatment groups, with a SMD of −1.01 (95% CI −2.77 to 0.74; *p* = 0.26).

##### Complications from injury and treatment

Skin or pressure-related complications were reported in all five studies comparing boot/brace and POP cast immobilisation devices.^4,13–15,18^ None of these studies or pooled analysis identified a significant difference between treatment groups (RR 0.91, 95% CI 0.28 to 2.93; *p* = 0.87).

##### Return to baseline ADL

One study reported return to ADL at 2 weeks follow-up using the Activity Scale for Kids performance scale (ASK-p) score.^15,21^ There was no significant difference in mean ASK-p scores between ankle brace and casted splint at 2 weeks (60.0 vs 54.2; *p* = 0.3103).

Four trials comparing boot/brace and cast immobilisation devices reported return to ADL (Figure 4).^4,13–15^ ADL scores were significantly higher in boot/brace groups in three studies (75.0%), whereas Barnett *et al* (25.0%) reported similar ADL scores between boot/brace and cast groups. Pooled analysis identified a significant difference between treatment groups, in favour of removable boots/braces, with a SMD of −0.79 (95% CI −1.03 to −0.55; *p* < 0.00001).

**Figure 4:**
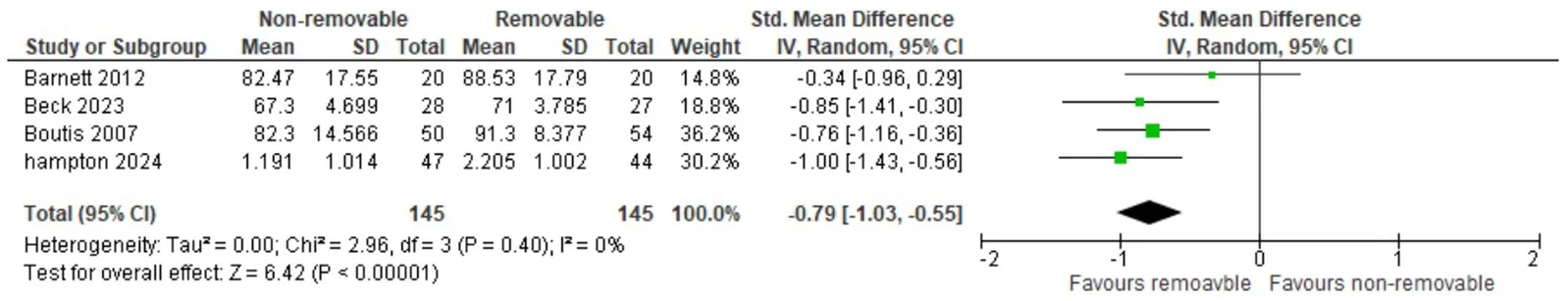
Forest plot comparing return to baseline activities of daily living between boot/brace and casted device groups.

Barnett *et al* reported the number of days before children returned back to their normal levels of activity.^15^ This study found that children treated with an ankle brace returned to normal activity levels significantly earlier than those treated with a casted splint, with a MD of −6.83 days (95% CI −11.20 to −2.96; *p* = 0.0031).

In addition, Boutis *et al* and Marson *et al* reported return to normal activities at 4- and 6-week follow-up, respectively (Figure 5).^4,18^ In both trials, a smaller proportion of children treated with removable ankle braces failed to return to normal activities compared with those treated with non-removable casts. Although the difference was only statistically significant in Boutis *et al*, pooled analysis confirmed overall significance, with a RR of 0.45 (95% CI 0.25 to 0.82; *p* = 0.009).

**Figure 5:**
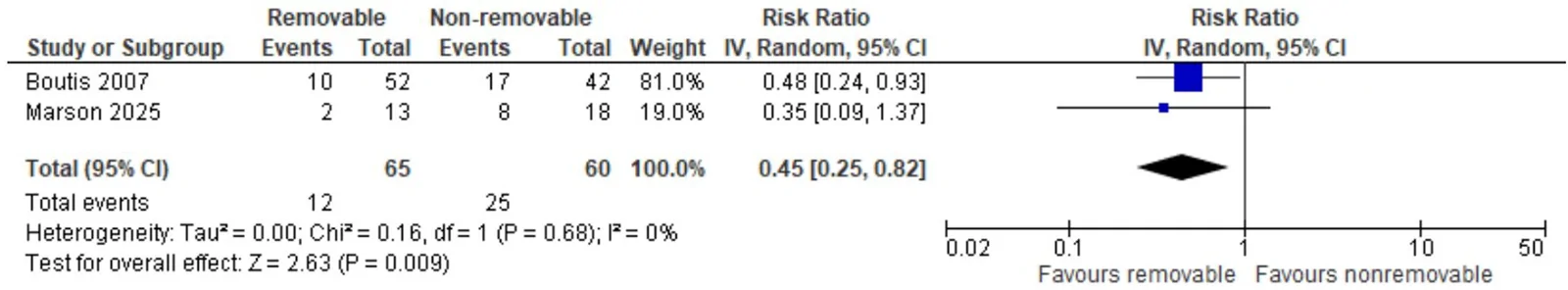
Forest plot comparing the proportion of patients returning to normal activities at 4–6-week follow-up between boot/brace and casted device groups.

##### Participation in learning: days off school

Participation in learning values, assessed by the mean number of school days children missed, were pooled from two studies.^13,18^ Pooled analysis showed no significant difference between removable boot/brace and non-removable cast groups (SMD −0.12, 95% CI −0.56 to 0.50; *p* = 0.59).

##### Mobility

One study reported the proportion children fully weight-bearing at 2-week follow-up,^15^ which found that a larger proportion of children treated with removable ankle braces were fully weight-bearing compared with children treatment with casted splints at 2-week follow-up (65% vs 40%). However, this difference was not significant (*p* = 0.1134). The proportion of children fully weight-bearing was also similar at 4-week follow-up (88% vs 95%; *p* = 0.4525). One study reported the proportion of patients weight-bearing with no pain at 4-week follow-up, finding no significant difference between brace and cast groups (72% vs 75%; *p* = 0.8993).^4^

One trial compared Proxy PROMIS mobility scores between patients treated with a removable ankle brace or non-removable cast.^18^ This study found a significant difference in favour of the ankle brace at 2 weeks (40.4 vs 34.8; *p* = 0.0206); however, mobility scores were similar at 6-week follow-up (49.2 vs 47.1; *p* = 0.4147).

##### Healthcare economic cost

One study reported the mean number of work hours missed by parents, finding no significant difference between work hours missed of parents whose children were treated with a removable boot compared with a fibreglass cast (4.07 vs 3.15; *p* = 0.53).^13^

Two trials reported estimated costs of treatment between the two treatment groups.^4,14^ Boutis *et al* estimated that overall, taking into account direct healthcare, parental work loss and other costs, treatment with removable ankle braces was significantly cheaper than treatment with a fibreglass cast (£150.98 vs £174.90), with healthcare costs found to be statically significant (*p* < 0.0001).^4^ Similarly, Hampton *et al* found treatment with removable walker boots to be significantly cheaper than cast treatment (£76.75 vs £180.83).^14^

##### Satisfaction

Two studies reported patient treatment satisfaction.^4,14^ Hampton *et al* reported outcomes for whether patients would want to receive the same treatment again and found that a significantly larger proportion of parents would want to receive walker boot treatment rather than a cast (93% vs 51%; *p* < 0.0001).^14^ Similarly, Boutis *et al* reported that significantly fewer patients were ‘not at all happy’ or ‘a little happy’ with their treatment device in the brace group compared with the cast group (11% vs 56%; *p* < 0.0001).^4^ In addition, this study found that whereas only 3 (5.7%) patients in the brace group reported cast preference, 27 (54%) patients in the cast group reported brace preference.

Three studies reported the proportion of parents who would want to receive the same treatment again.^4,14,15^ Although Barnett *et al* reported similar satisfaction between parents of children treated with ankle braces and casted splints (92% vs 90%), Hampton *et al* found that parent satisfaction mirrored patient satisfaction in favour of removable boot treatment (89% vs 55%; *p* < 0.0005).^14,15^ Similarly, Boutis *et al* found that parental satisfaction, measured using a 4-point Likert scale, was significantly higher in the brace group than in the cast group (*p* < 0.0001).

##### Missing outcomes

None of the five trials comparing removable boot/brace with non-removable casted devices reported on appearance and deformity or time to union.

## Discussion

We found that that seven RCTs investigated the use of different immobilisation devices for the treatment of low-risk, stable or occult ankle fractures, with three studies comparing the use of soft (functional) immobilisation with functional immobilisation devices and five studies comparing removable boots/braces with casted devices. This systematic review has more trials than previous reviews, enabling these subgroup analyses to be conducted.

However, the ability to make definitive conclusions between soft and rigid immobilisation is limited by the considerable heterogeneity across included studies. The primary sources of heterogeneity for this comparison were as follows:

- Study participants: the three trials comparing soft and rigid immobilisation devices had differing populations of children, with mean ages ranging from 9 to 14 years.
- Included injuries: Marson *et al* clearly defined their inclusion criteria, only including clinically observable fractures.^18^ However, the other two trials were less specific in their inclusion criteria, potentially including more simple sprains that would resolve without intervention.^11,12^ This variation may bias results in favour of the less restrictive soft immobilisation treatment strategy.
- Treatment strategies: each study used different immobilisation devices and treatment regimens. Soft immobilisation varied between trials, with supportive bandaging used in two studies, whereas Launay *et al* used no immobilisation.^11,12,18^ For rigid immobilisation, two studies directly used solid immobilisation with plaster.^11,12^ Conversely, the Marson *et al* trial, which had three arms in total, included two rigid immobilisation devices (synthetic cast and semi-rigid ankle brace).^18^ Whereas cast groups from all three trials restrict ankle dorsiflexion and plantarflexion, the ankle brace group allowed some degree of motion, potentially influencing comfort, mobility and recovery.
- Outcome measures: there was significant inconsistency in reported outcomes. None of the three trials reported the primary outcome of unscheduled returns to a healthcare facility, or several key outcomes from the core outcome set including return to physical and recreational activities, appearance and deformity or time to union. Only two studies used previously validated patient-reported measures: Launay *et al* for pain and quality of life and Marson *et al* for quality of life and mobility.^11,18^
- Power and sample size: of the three trials, only Launay *et al* performed a prospective sample size calculation.^11^ Marson *et al* was a feasibility trial and was therefore not designed or powered to detect treatment effects.^18^

However, although heterogeneity was present in the boot/brace vs casted device comparison, this was lower than in the soft vs rigid immobilisation comparison. The most important sources of heterogeneity for the boot/brace vs cast immobilisation comparison were as follows:

- Study participants: the five trials comparing removable boots/braces with casted materials has less variation in patient age, with mean ages varying between 10 and 11.4 years.
- Included injuries: all five trials had clear inclusion criteria and included children with similar diagnoses (eg SH1 distal fibula fractures and avulsion fractures).
- Treatment strategies: for the boot/brace groups, three studies used a similar brace, and two studies used a boot.^4,13,15,18^ However, casted immobilisation devices were more varied between studies, including synthetic casts, fibreglass casts, removable splints.^4,13–15,18^
- Outcome measures: four of the five trials reported the primary outcome of unscheduled returns to a healthcare facility and four studies reported outcomes using previously validated patient-reported measures: validated pain scores, ADL using ASK-p, and emotional or overall health/quality of life, each reported in two studies.^4,13–15^
- Power and sample size: three of the five trials did not conduct a prospective sample size calculation so may be underpowered.^13,14,18.^

Despite these limitations, there is strong evidence to suggest that removable boots/braces are preferable to casted devices for managing these injuries. Pooled analysis demonstrates that treatment with removable boots/braces was associated with a significantly lower rate of unscheduled returns to hospital for additional follow-up and significantly improved return to baseline ADL compared with treatment with casted immobilisation devices. In addition, it was found that removable boots/braces provided superior patient and parent satisfaction compared with casted immobilisation devices. There was no significant difference for pain, emotional/overall health, complications or participation in learning.

The significantly improved return to ADL at 4–6-week follow-up, and superior function as measured by the validated ASK-p score in patients treated with removable boots/braces compared with casted devices is likely to be significant for patients and their families. This is reflected by significantly superior satisfaction. In addition, with the rising need for cost-effective treatments, the reduced need for unexpected returns to healthcare facilities and healthcare economic cost is significant. These findings are consistent with the conclusions of previous systematic reviews. A Cochrane review by Yeung *et al* in 2016, which included three RCTs involving 207 children, reported that treatment with removable ankle braces enables faster functional recovery than treatment with rigid cast immobilisation.^22^ This highlights the benefit in providing treatment that permits ankle dorsiflexion and plantarflexion in optimising patient recovery and return to ADL. Our original systematic review of four trials also suggested this benefit, but was limited by a lack of high-quality evidence.^5^ Interestingly, a recent systematic review of adult ankle fracture immobilisation by Zhou *et al* reported similar findings, concluding that by enabling some movement at the ankle joint during the immobilisation period, removable ankle braces provide superior early and mid-term functional outcomes, and reduce the risk of deep vein thrombosis, compared with cast immobilisation.^23^

However, the necessity for rigid immobilisation compared with functional immobilisation remains uncertain. Pooled analysis showed that children treated with soft immobilisation missed significantly fewer days of school, a difference that is meaningful for patients and families. In addition, soft immobilisation was associated with a significantly faster return to normal function and fewer missed work hours for parents compared with children treated with rigid immobilisation devices. The 6-day improvement in return to normal activities is likely to be relevant to families. However, the small difference for school days missed and the fact that these outcomes were reported in only a limited number of trials limits the strength of these findings and highlights the need for further investigation. No significant difference was identified between soft and rigid immobilisation devices for pain, emotional/overall health or mobility.

Other limitations of this systematic review include the small sample sizes and short follow-up periods of included trials, with many failing to report key outcomes defined by the core outcome set for childhood limb fractures.^19^ In addition, the lack of any format cost-effectiveness analysis limits the ability to conduct an economic comparison of treatments. Larger RCTs are required to determine whether rigid immobilisation is necessary for these fractures. These trials should have a sufficiently long follow-up, assess outcomes aligned with the established core outcome set for childhood limb fractures, and include a formal cost-effectiveness analysis to inform evidence-based treatment guidelines.

## Conclusion

There is some evidence that treatment with removable boots/brace immobilisation devices offers advantages over immobilisation with casted devices in the management of low-risk, stable or occult ankle fractures in children. In this systematic review, we found that removable ankle boots/braces were associated with fewer unscheduled hospital visits and provided superior functional outcomes, as well as higher patient and parent satisfaction. However, there is no evidence supporting the necessity for rigid immobilisation. This systematic review found that functional immobilisation with supportive bandages improves participation in learning and reduces missed parental work hours, without any significant differences in pain, emotional/overall health, or satisfaction compared with rigid immobilisation devices.

## Competing interests

The author/s declare no competing interests.

## Funding

The author/s received no financial support for the research, authorship and/or publication of this article.

## Artificial Intelligence

The author/s declare that no AI was used to conduct the study or prepare the manuscript.

## References

[1] Venkatadass K, Sangeet G, Prasad VD, Rajasekaran S. Paediatric ankle fractures: guidelines to management. *Indian J Orthop* 2021; **55**: 35–46. 10.1007/s43465-020-00270-9 PMC7851308 33569097

[2] Bible JE, Smith BG. Ankle fractures in children and adolescents. *Tech Orthop* 2009; **24**: 211–219.

[3] Su AW, Larson AN. Pediatric ankle fractures: concepts and treatment principles. *Foot Ankle Clin* 2015; **20**: 705–719. 10.1016/j.fcl.2015.07.004 PMC4912125 26589088

[4] Boutis K, Willan AR, Babyn P *et al.* A randomized, controlled trial of a removable brace versus casting in children with low-risk ankle fractures. *Pediatrics* 2007; **119**: e1256–e 12 63. 10.1542/peds.2006-2958 17545357

[5] Marson BA, Ng J, Myint Y *et al.* Management of ‘low-risk’ ankle fractures in children: a systematic review. *Ann R Coll Surg Engl* 2019; **101**: 539–545. 10.1308/rcsann.2019.0027 PMC6818065 30855167

[6] Flynn JM, Skaggs D, Sponseller PD *et al.* The operative management of pediatric fractures of the lower extremity. *J Bone Joint Surg* 2002; **84**: 2288–2300.

[7] Boutis K, Howard A, Constantine E *et al.* Evidence into practice: emergency physician management of common pediatric fractures. *Pediatr Emerg Care* 2014; **30**: 462–468. 10.1097/PEC.0000000000000162 24977995

[8] Beck JJ, Carpenter CM, West N *et al.* Treatment variability and complications associated with pediatric lateral ankle injuries: a POSNA quality, safety, and value initiative survey. *Orthop J Sports Med* 2022; **10**: 23259671221100223. 10.1177/23259671221100223 PMC9163735 35668871

[9] Tay JY, Lee NKL, Chou ACC *et al.* Non-surgical interventions for isolated Salter–Harris type I distal fibula fractures: a systematic review. *J Orthop Rep* 2024; **3**: 100319.

[10] Shirley ED, Maguire KJ, Mantica AL, Kruse RW. Alternatives to traditional cast immobilization in pediatric patients. *J Am Acad Orthop Surg* 2020; **28**: e20–e27. 10.5435/JAAOS-D-18-00152 31290757

[11] Launay F, Barrau K, Simeoni M *et al.* Ankle injury without fracture in children: cast immobilization versus symptomatic treatment. Impact on absenteeism and quality of life. *Arch Pediatr* 2008; **15**: 1749–1755. 10.1016/j.arcped.2008.09.008 18976892

[12] Gleeson A, Stuart M, Wilson B, Phillips B. Ultrasound assessment and conservative management of inversion injuries of the ankle in children: plaster of Paris versus Tubigrip. *J Bone Joint Surg Br* 1996; **78**: 484–487. 8636192

[13] Beck JJ, Kang V, Bennett A *et al.* Controlled ankle movement (CAM) boot provides improved outcomes with lower complications than short leg walking cast in low-energy pediatric lateral ankle injuries: a prospective, randomized study. *J Pediatr Orthop* 2023; **43**: 418–423. 10.1097/BPO.0000000000002425 37130811

[14] Hampton MJ, Hilton C, Nicolaou N, Flowers MJ. Functional walker boots are preferred to synthetic casts by patients and carers in the management of pediatric stable ankle injuries. *J Pediatr Orthop* 2024; **44**: 99–105. 10.1097/BPO.0000000000002570 37982460

[15] Barnett PLJ, Lee MH, Oh L *et al.* Functional outcome after air-stirrup ankle brace or fiberglass backslab for pediatric low-risk ankle fractures: a randomized observer-blinded controlled trial. *Pediatr Emerg Care* 2012; **28**: 745–749. 10.1097/PEC.0b013e318262491d 22858744

[16] Abzug JM, Schwartz BS, Johnson AJ. Assessment of splints applied for pediatric fractures in an emergency department/urgent care environment. *J Pediatr Orthop* 2019; **39**: 76–84. 10.1097/BPO.0000000000000932 28060178

[17] Ceroni D, Martin X, Delhumeau C *et al.* Effects of cast-mediated immobilization on bone mineral mass at various sites in adolescents with lower-extremity fracture. *J Bone Joint Surg Am* 2012; **94**: 208–216. 10.2106/JBJS.K.00420 22298052

[18] Marson BA, Gurney M, Manning JC *et al.* Supportive bandage, removable splint, or walking casts for low-risk ankle fractures in children: a feasibility randomized controlled trial. *Bone Joint J* 2025; **107**: 108–117. 10.1302/0301-620X.107B1.BJJ-2024-0354.R1 39740682

[19] Marson BA, Manning JC, James M *et al.* Development of the CORE-kids core set of outcome domains for studies of childhood limb fractures. *Bone Joint J* 2021; **103-b**: 1821–1830. 10.1302/0301-620X.103B.BJJ-2020-2321.R2 PMC8779948 34412506

[20] Sterne JAC, Savović J, Page MJ *et al.* Rob 2: a revised tool for assessing risk of bias in randomised trials. *BMJ* 2019; **366**: l4898. 10.1136/bmj.l4898 31462531

[21] Young NL, Williams JI, Yoshida KK, Wright JG. Measurement properties of the activities scale for kids. *J Clin Epidemiol* 2000; **53**: 125–137. 10.1016/s0895-4356(99)00113-4 10729684

[22] Yeung DE, Jia X, Miller CA, Barker SL. Interventions for treating ankle fractures in children. *Cochrane Database Syst Rev* 2016; **4**: Cd010836. 10.1002/14651858.CD010836.pub2 PMC7111433 27033333

[23] Zhou S, Zheng H, Cao M *et al.* Comparative efficacy of cast immobilization versus removable braces in patients with ankle fractures: a systematic review and meta-analysis. *BMC Musculoskelet Disord* 2025; **26**: 243. 10.1186/s12891-025-08451-z PMC11895275 40069691

